# Efficacy of Suxiao Jiuxin Pill on Coronary Heart Disease: A Meta-Analysis of Randomized Controlled Trials

**DOI:** 10.1155/2018/9745804

**Published:** 2018-03-27

**Authors:** Li Ren, Jie Wang, Ling Feng, Shuli Wang, Jun Li

**Affiliations:** Department of Cardiovascular, Guang'anmen Hospital, China Academy of Chinese Medical Sciences, Beijing 100053, China

## Abstract

Suxiao jiuxin pill is considered an effective ancillary drug in patients with coronary heart disease. Although numerous small, single-center clinical trials have been conducted, the benefits and harms of suxiao jiuxin pill remain controversial. We performed a meta-analysis to clarify the efficacy of suxiao jiuxin pill on patients with coronary heart disease. Randomized controlled trials were identified by using the Cochrane Library, PubMed, Web of Science, Embase, Wanfang, Weipu, and China Knowledge Resource Integrated databases (until June 2016). Pooled relative risks (RR), weighted mean differences (WMD), and 95% confidence intervals (95% CIs) were estimated using random-effects models. Forty-one trials involving 6276 patients were included in our analysis. Administration of suxiao jiuxin pill significantly improved electrocardiogram (ECG) results when compared with other therapies (RR 1.32, 95% CI 1.26 to 1.38, and *P* < 0.001). Subgroup analyses revealed that suxiao jiuxin pills improve ECG results more than salvia tablets (RR 1.54, 95% CI 1.41 to 1.67, and *P* < 0.001), isosorbide dinitrate (RR 1.14, 95% CI 1.21 to 1.44, and *P* = 0.001), nitroglycerin (RR 1.35, 95% CI 1.16 to 1.56, and *P* < 0.001), and other drugs (RR 1.32, 95% CI 1.21 to 1.44, and *P* < 0.001). Available evidence additionally suggests that suxiao jiuxin pills could significantly reduce total cholesterol (WMD −0.62 mmol/L, 95% CI −1.06 to –0.18 mmol/L, and *P* = 0.005) and low-density lipoprotein (LDL) levels (WMD −1.12 mmol/L, 95% CI −1.42 to −0.82 mmol/L, and *P* < 0.001) and increase high-density lipoprotein (HDL) levels (WMD 0.32 mmol/L, 95% CI 0.07 to 0.58 mmol/L, and *P* = 0.014). However, no significant differences were observed in total triglyceride levels, plasma viscosity, hematocrit, and fibrinogen. No incidences of adverse reactions were observed after administration of suxiao jiuxin pill. Improvements in ECG results and lipid profiles were also observed after suxiao jiuxin administration compared to other therapies. It also decreased low-cut and high-cut whole blood viscosity without significant adverse reactions.

## 1. Introduction

Coronary heart disease (CHD) has become the leading cause of death in both men and women worldwide [1]. Most CHD-related deaths occur in individuals older than 65 years of age. The spectrum of CHD includes subclinical CHD, chronic stable angina pectoris, unstable angina, and acute myocardial infarction. A large number of elderly patients have asymptomatic heart disease; therefore, the prevalence of CHD may be underestimated [2]. Several large prospective clinical studies [3–7] have demonstrated that CHD is significantly associated with atrial fibrillation, congestive heart failure, stroke, and other serious diseases. Hence, it is important to develop effective therapies to mitigate the progression of this disease.

Suxiao jiuxin pills are one of the most commonly used Chinese medicines for cardiocerebral vascular conditions. They were first developed by Chinese medicine specialist Chenggui Zhang in the 1980s and manufactured by the Sixth Chinese Drugs Factory of Tianjin Zhongxin Pharmaceutical Co., Ltd. [8, 9]. Small doses of suxiao jiuxin pill have been shown to rapidly relieve angina pectoris and improve its symptoms without any obvious side effects. Several reports have suggested that suxiao jiuxin pill helps lower the patients' lipid profile and improve myocardial function [10]. The main components of suxiao jiuxin pills are borneol and* Ligusticum chuanxiong* Hort [9, 11–15]. These ingredients can effectively induce relaxation and inhibit artery contraction [9, 11]. In addition, several smaller clinical studies have been conducted to study the efficacy of suxiao jiuxin pill on CHD patients; however, these results have been inconsistent [16–19]. Cao and Zhang suggested that suxiao jiuxin pill was associated with symptom remission, reduced incidence of angina, and shorter duration of angina. Further, electrocardiogram results were significantly improved by nitroglycerin use compared with salvia [16]. Qiao et al. demonstrated that suxiao jiuxin pills plus trimetazidine therapy significantly reduced the effective rate of angina, but other relevant indices were not evaluated [17–19]. Clarifying the beneficial and harmful effects of suxiao jiuxin pill is particularly important for CHD patients, as they have not been distinctly determined with respect to ECG results, lipid profiles, hemorheology, and adverse reactions. Therefore, we performed a large-scale meta-analysis of the available randomized controlled trials to determine the benefits of suxiao jiuxin pill for CHD patients.

## 2. Materials and Methods

Ethical approval and written consent were not necessary for the meta-analysis, as the data was collected from published literature.

Our meta-analysis was conducted according to the Preferred Reporting Items for Systematic Reviews and Meta-Analyses guidelines [20]. We searched the Cochrane Library, PubMed, Web of Science, Embase, Wanfang, Weipu, and China Knowledge Resource Integrated databases to identify relevant studies published in English or Chinese prior to June 2016. Our search terms included “coronary heart disease,” “suxiao jiuxin pill,” and “coronary artery disease.” We also searched for meta-analysis publications and bibliographies referenced in the selected publications. Gray literature was identified through related agencies and clinical trial registers. Clinical trials that compared the efficacy of suxiao jiuxin pill on coronary heart disease with those of placebo or standard therapy were included in this meta-analysis. Criteria for inclusion were as follows: (1) a randomized controlled study design, (2) the possibility of extracting accurate clinical data, (3) classifying coronary heart disease based on the updated guidelines of the American Heart Association and American College of Cardiology Foundation [21, 22], and (4) reporting ECG results, lipid profiles, and/or hemorheology changes as outcomes. Two reviewers (X. L. H. and J. Z. J.) independently reviewed the studies to determine whether they satisfied the eligibility criteria. Discrepancies between reviewers' opinions were resolved by consensus, and a third reviewer was consulted when necessary.

### 2.1. Data Extraction

Two independent reviewers using the same checklist evaluated the data from the included studies. Disagreements between the reviewers were resolved by discussion until consensus was reached. The following sets of data were extracted for each selected study when available: demographics and sample characteristics, definition of coronary heart disease, and usage of suxiao jiuxin pill. The primary outcome of the selected studies was the improvement in ECG results, including resting ECG returning to normal or negative submaximal exercise test. The secondary outcomes included changes in lipid profile (total cholesterol, total triglyceride, low-density lipoprotein, and high-density lipoprotein) and hemorheology (high-cut whole blood viscosity, low-cut whole blood viscosity, plasma viscosity, hematocrit, and fibrinogen) as well as any adverse reactions.

### 2.2. Quality Assessment

Two reviewers independently assessed the methodological quality of the studies using the Jadad scoring system [23]. Five aspects for each study were thoroughly evaluated: the statement of randomization, the method used for generating randomized sequences for treatment assignments, the use of double-blind design, the description of the double blinding method, and data on withdrawals and dropouts. Studies with a score less than 3 were considered as low quality studies with high bias risk. Studies that received a score of 3 or greater were considered as high-quality studies. Disagreements between the reviewers were resolved by consensus and consultation with a third reviewer when necessary.

### 2.3. Data Analysis

Continuous variables, such as changes in lipid profiles and hemorheology, were expressed as mean ± standard deviation. Categorical data, such as ECG result improvement and adverse reaction incidence, were presented as frequencies and percentages. We computed the pooled relative risk (RR), weighted mean difference (WMD), and 95% confidence interval (CI), as well as the heterogeneity of the included studies by using random-effect (DerSimonian and Laird) models. Metaregression analysis was conducted based on sample size and mean age to explore the impact of sample size on the source of heterogeneity [24]. We also performed subgroup analyses to compare the efficacy of different drugs with those of suxiao jiuxin pill on ECG result improvement. Heterogeneity was quantified using the *I*^2^ statistic. We considered *I*^2^ values greater than 50% to indicate significant heterogeneity between the studies. Statistical heterogeneity between studies was also formally tested with the Cochran test (*P* < 0.10) [25, 26]. Publication bias was evaluated using the funnel plot and Egger's and Begg tests, with *P* values less than 0.05 considered significant publication bias. Two-tailed *P* values less than 0.05 were considered statistically significant. All statistical analyses were performed with STATA 12.0 (Stata Corporation, College Station, TX, USA).

## 3. Results

### 3.1. Search Results

**Figure 1:**
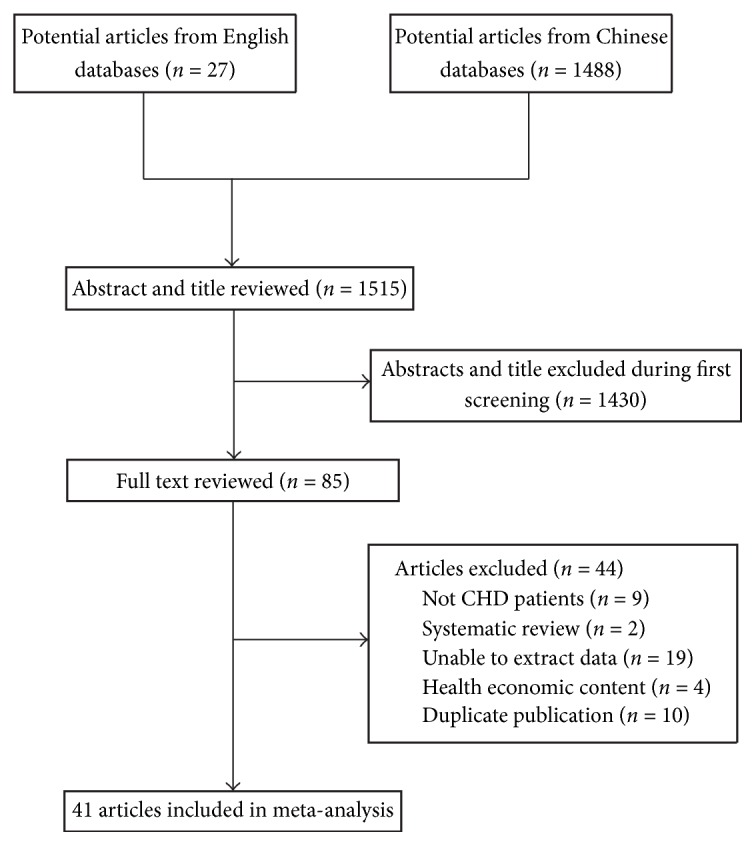
Process used to select relevant studies for inclusion in the meta-analysis.

The search strategy revealed 1515 potentially eligible publications. After duplicate removal, 1253 studies remained. Abstracts were evaluated based on the inclusion and exclusion criteria. 85 studies warranting further review were identified. Among these, 44 studies were excluded for the reasons listed in Figure 1. The remaining 41 studies were included in our meta-analysis. Journal articles and full manuscripts were obtained for all 41 studies.

### 3.2. Study Characteristics

The characteristics of the trials included in our meta-analysis are presented in Table 1. All of the included studies were conducted in China. The following studies were included as the control group: 2 studies involving standard treatment [27, 28], 7 studies involving nitroglycerin (using various formulations) [29–35], 12 studies involving isosorbide dinitrate [36–47], 11 studies involving salvia tablets [16, 17, 48–56], and 9 studies involving Chinese herbal pills other than suxiao jiuxin [18, 32, 57–64].

The 41 studies consisted of 6276 patients with coronary heart disease. The mean age in the treatment group was 57.57 ± 8.15 years and 54.10% of the patients were male. The mean age in the control group was 57.80 ± 8.72 years and 45.96% of the patients were male. The baseline characteristics were balanced between the treatment and control groups. The majority of the included studies received low Jadad scores due to the lack of a double-blind design (Table 2).

### 3.3. ECG Result Improvement

**Figure 2:**
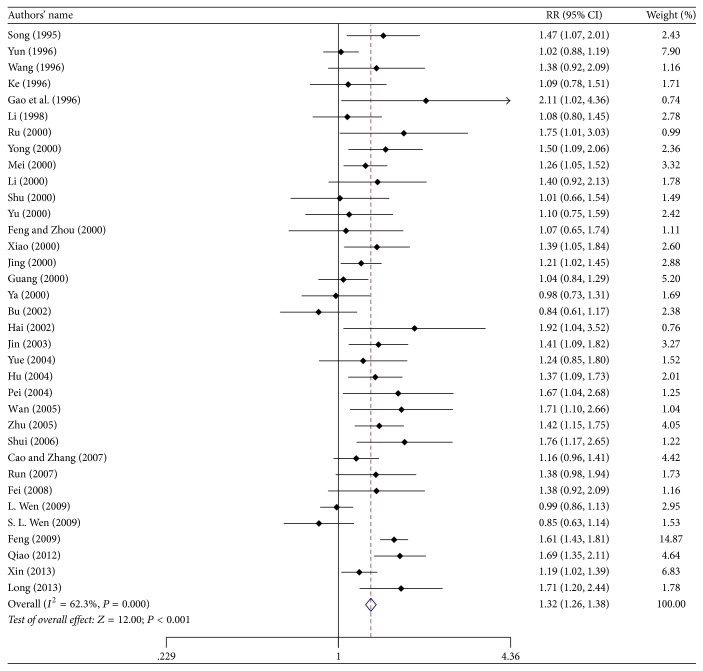
Relative risks for ECG improvement in the treatment and control groups.

**Figure 3:**
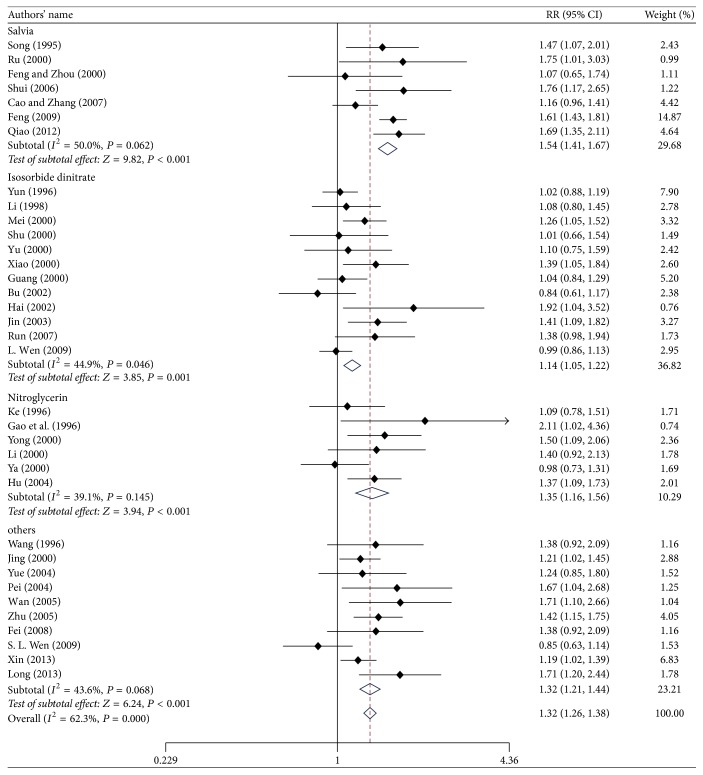
Relative risks for ECG improvement in the various subgroups.

**Figure 4:**
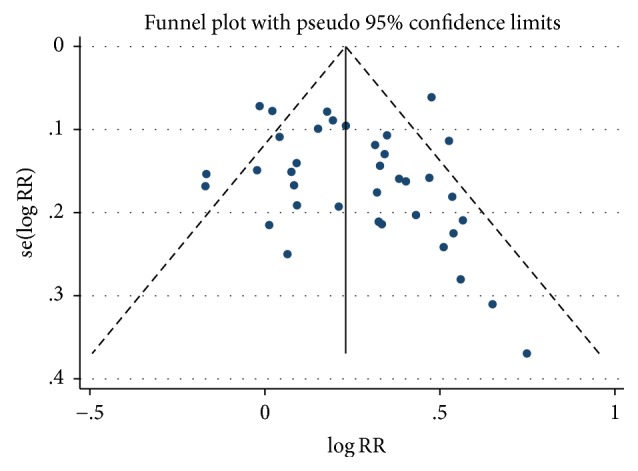
Funnel plot of studies included in the meta-analysis.

Figure 2 presents the results of the meta-analysis of ECG improvement following administration of suxiao jiuxin pills. ECG results were reported in 35 studies. Pooled analysis indicated significant benefits of suxiao jiuxin pill on ECG outcomes (RR 1.32; 95% CI 1.26 to 1.38, *P* < 0.001). However, there was significant heterogeneity between studies with respect to ECG results (*I*^2^ = 62.3%). Findings from the meta-regression analyses suggested that sample size and mean age of the patients were not significant factors contributing to the association between suxiao jiuxin pills and ECG outcomes (Table 3). Considering that the control group may be the source of the heterogeneity, we performed subgroup analyses to compare the effects of suxiao jiuxin pill with those of control treatment (Figure 3). The subgroup analyses indicated that suxiao jiuxin pill improved ECG results more than salvia tablets (RR 1.54; 95% CI 1.41 to 1.67, *P* < 0.001), isosorbide dinitrate (RR 1.14; 95% CI 1.05 to 1.22, *P* = 0.001), nitroglycerin (RR 1.35, 95% CI 1.16 to 1.56, *P* < 0.001), and other drugs (RR 1.32, 95% CI 1.21 to 1.44, *P* < 0.001). The *I*^2^ decreased to 39.1% in the nitroglycerin subgroup. However, moderate heterogeneity was still observed among the other three subgroups (*I*^2^ = 50.0%, 44.9%, and 43.6%, resp.). Sensitivity analysis was conducted by excluding individual studies one after another but did not reveal a substantial change in the overall trend of heterogeneity between studies. We also constructed a funnel plot to assess the degree of publication bias. The funnel plot was symmetrically distributed around the pooled effect size, which indicated the absence of significant publication bias in the included studies (Figure 4). In addition, examining the funnel plot asymmetry via Egger test (*P* = 0.067) and Begg test (*P* = 0.050) did not demonstrate publication bias.

### 3.4. Lipid Profile

Only four of the studies reported the efficacy of suxiao jiuxin pill on patients' lipid profiles, and they all reported the total cholesterol in the patients' serum [33, 55, 57, 63]. Significant lower total cholesterol levels were reported in the suxiao jiuxin pill group compared with the control group (WMD −0.62 mmol/L, 95% CI −1.06 to –0.18 mmol/L, and *P* = 0.005). There was significant heterogeneity among the 4 studies with respect to total cholesterol levels (*I*^2^ = 77.1%) (Supplemental Figure S1). In addition, the four studies reported total triglyceride levels in the patients' serum [33, 55, 57, 63]. There was no significant difference in WMD between the treatment and control groups (WMD −0.59 mmol/L, 95% CI −1.72 to 0.54 mmol/L, and *P* = 0.303). We detected significant heterogeneity between studies among the 4 studies with respect to total triglyceride levels (*I*^2^ = 98.2%) (Supplemental Figure S2). Three of the studies reported LDL levels [33, 57, 63]. There were significantly lower LDL levels in the treatment group compared with the control group (WMD −1.12 mmol/L, 95% CI −1.42 to −0.82 mmol/L, and *P* < 0.001) and lower heterogeneity between the 3 trials (*I*^2^ = 56.5%) (Supplemental Figure S3). Furthermore, the four studies reported HDL levels [30, 52, 54, 60]. There were significantly higher HDL levels in the treatment group compared with the control group (WMD 0.32 mmol/L, 95% CI 0.07 to 0.58 mmol/L, and *P* = 0.014), however with significant heterogeneity with respect to HDL levels (*I*^2^ = 87.5%) (Supplemental Figure S4). Sensitivity analysis did not reveal any single study as the source of heterogeneity. We did detect sample size (*P* = 0.013) as a contribution to the association between suxiao jiuxin pill and total triglyceride level, with no other significant factors being observed (Table 3).

### 3.5. Hemorheology

Six studies reported the levels of low-cut whole blood viscosity after treatment [39, 41, 43, 53, 57, 63]. There were significantly lower low-cut whole blood viscosity levels in the treatment group compared to the control group (WMD −1.57 mpa·s, 95% CI −2.50 to −0.65 mpa·s, and *P* = 0.001). Significant heterogeneity was observed among these studies with respect to the level of low-cut whole blood viscosity (*I*^2^ = 86.8%) (Supplemental Figure S5). Six studies reported the levels of high-cut whole blood viscosity [39, 41, 43, 53, 57, 63], which were significantly lower in the treatment group (WMD −0.69 mpa·s, 95% CI −1.03 to −0.34 mpa·s, and *P* < 0.001). Significant heterogeneity was also observed among these studies with respect to the level of high-cut whole blood viscosity (*I*^2^ = 87.6%) (Supplemental Figure S6). Seven studies reported the levels of plasma viscosity [33, 39, 41, 43, 54, 57, 63]. There was no significant difference in WMD between the treatment and control groups (WMD −0.03 mpa·s, 95% CI −0.07 to 0.01 mpa·s, and *P* = 0.186) and no significant heterogeneity between studies among these studies with respect to the level of plasma viscosity (*I*^2^ = 4.9%) (Supplemental Figure S7). Four studies reported the levels of hematocrit [33, 54, 57, 63] and no significant differences were observed in WMD between the treatment and control groups (WMD −1.24%, 95% CI −3.26 to 0.77%, and *P* = 0.227). There was significant heterogeneity among three studies with respect to the level of hematocrit (*I*^2^ = 84.3%) (Supplemental Figure S8). Four studies reported the levels of fibrinogen [33, 53, 57, 63] with no significant difference in WMD between the treatment and control groups (WMD −0.76 g/L, 95% CI −1.32 to −0.20 g/L, and *P* = 0.008). There was significant heterogeneity among the three studies with respect to the fibrinogen level (*I*^2^ = 85%) (Supplemental Figure S9). Sensitivity analysis was conducted by excluding each study individually and showed no substantial change in the overall trend. Furthermore, sample size and mean age were not correlated with treatment efficacy of suxiao jiuxin pills on hemorheology (Table 3).

### 3.6. Adverse Reactions

Fourteen studies reported the incidence of adverse reactions. The most common symptoms were mild headache, dizziness, and facial flushing. Most of these symptoms resolved spontaneously. There was no significant difference in the adverse reaction rates between the treatment and the control groups (RR 1.12, 95% CI 0.50 to 2.51, and *P* = 0.785) and no significant heterogeneity with respect to adverse reaction rate (*I*^2^ = 49.1%) (Supplemental Figure S10). Additionally, meta-regression analyses suggested that both sample size and mean age were not associated with adverse reaction in the suxiao jiuxin pill treatment group (Table 3). The funnel plot was symmetrically distributed around the pooled effect size, which indicated the absence of significant publication bias in the included studies (Supplemental Figure S11). In addition, no publication bias was identified using the Egger test (*P* = 0.064) or Begg test (*P* = 0.274).

## 4. Discussion

Based on our meta-analysis, we found that suxiao jiuxin pills could significantly improve ECG results in CHD patients compared with other therapies used in the selected studies. Suxiao jiuxin pills decreased the levels of total cholesterol and LDL, increased the levels of HDL, and lowered low-cut and high-cut whole blood viscosity. Other hemorheology-related parameters, such as plasma viscosity, hematocrit, and fibrinogen, showed the same tendency, but these changes were not statistically significant.

In recent years, several randomized clinical trials have been performed to evaluate the efficacy of suxiao jiuxin pill on CHD patients [17, 27, 28, 56]. Long studied 120 patients with unstable angina and found that suxiao jiuxin pills significantly improved both ECG results and symptoms compared with standard treatment [28]. Bu evaluated the benefits of suxiao jiuxin pills with isosorbide dinitrate on one hundred coronary heart disease (CAD) patients [44]. However, they did not find any significant differences between the groups with respect to ECG result improvement and angina relief. This may be due to the small sample size, different inclusion criteria, and differences in treatment strategy.

Two previous meta-analyses that explored the efficacy of suxiao jiuxin pills on CHD [65, 66] found them to be effective in the treatment of angina pectoris, without any serious side effects. However, due to the limited sample sizes, low quality of the studies, and other potential confounding factors, the asymmetry funnel plot demonstrated the lack of reliability of these meta-analyses [65, 66]. As a result, we conducted this updated meta-analysis of randomized controlled trials to further clarify the effects of suxiao jiuxin pills on CHD patients.

Suxiao jiuxin pills have been widely used in China for many years in patients with angina. It has two main effective components, borneol and* Ligusticum chuanxiong* Hort, which can be found mainly in the Sichuan province of China.* Ligusticum chuanxiong* Hort was first described in the 'Divine Husbandman's Materia Medica.'* L. chuanxiong* has long been regarded as a traditional Chinese medicine and has been added to food for its health benefits. The main chemical components of* L. chuanxiong* include essential oils, phenolic acids, and phthalide lactones [67, 68]. Several researchers have demonstrated that* L. chuanxiong* could lower serum cholesterol and lipoprotein levels, reduce red blood cell deformability, and relieve angiotensin II–induced vascular smooth muscle cell proliferation. These unique roles may be due to the increase in nitric oxide and suppression of nuclear factor-*κ*B activation [69, 70]. In addition,* L. chuanxiong* has a direct vasodilatory effect on isolated aortic rings in rats [71]. The mechanisms of this effect are related to the opening of SK (Ca) and ATP_K_ channels, the reduction of ET-1, and the formation of reactive oxygen species (ROS) [72, 73]. Recent evidence has suggested that* L. chuanxiong* may exert antiplatelet effects by inhibiting the vWF-mediated process of platelet thrombus formation.

Borneol is a fragrant ingredient used in decorative cosmetics and is widely regarded as an adjuvant in Chinese herbs [74]. Several animal studies have demonstrated that borneol can dilate coronary arteries and improve coronary circulation. In addition, studies have demonstrated that borneol can inhibit the inflammatory response in animal models [75]. As a result, borneol is widely used in China for the treatment of CHD patients in clinical practice.

We found that the main side effects of suxiao jiuxin pills were mild headaches, dizziness, and facial flushing, all of which resolved spontaneously. The adverse reaction incidence associated with suxiao jiuxin pill was not significantly different from those associated with other treatments. Evidence suggests that suxiao jiuxin pills are safe with no adverse effects.

As in many meta-analyses, there were several limitations to our study. Most of the clinical studies included in our meta-analysis were of poor quality based on their Jadad scores. Only a few studies reported detailed research methodology, a factor that could decrease the reliability of this meta-analysis. In addition, all of the included studies were from Chinese publications, and this may be a source of bias. Significant heterogeneity between studies was noted in our meta-analysis even after subgroup analysis. Different study quality, sample size, usage of suxiao jiuxin pill, and control groups may contribute to this heterogeneity. In this study, we did not analyze cardiovascular death or drug-related complications.

In summary, our meta-analysis demonstrated that suxiao jiuxin pills improved ECG results and lipid profiles better compared with nitroglycerin, isosorbide dinitrate, salvia, and other Chinese herbal pills. They also decreased low-cut and high-cut whole blood viscosity.

## 5. Conclusions

Suxiao jiuxin pills can effectively decrease the lipid profiles and improve hemorheology parameters in CHD patients. This is due to the effects of their components (borneol and* L. chuanxiong*), which may improve coronary artery circulation and ECG results.

## Figures and Tables

**Table 1:** Characteristics of selected clinical trials included in the meta-analysis.

Author name	Year	Sample size	Study design	Age	Age	Country	Comparators	Dosage/frequency/cycle		Outcomes	Follow-up period
				(treatment)	(control)			Suxiao jiuxin pills	Comparators		
He	1995	48	RCT	42–60	40–57	China	Salvia	4# tid	3# tid	Hemorheology	4 weeks

Song	1995	149	RCT	55 ± 7.2	56 ± 6.9	China	Salvia	5# tid	3# tid	ECG, blood pressure, and heart rate	4 weeks

Wang	1996	60	RCT	NR	NR	China	Shexiang Baoxin pills	5# tid	3# tid	ECG and symptom	2 weeks

Ke	1996	72	RCT	45–79	46–78	China	Nitroglycerin	6# tid	0.5 mg Q8 h	ECG and symptom	4 weeks

Gao et al.	1996	147	RCT	36–85	35–84	China	Nitroglycerin	10#	0.5 mg	ECG and symptom	Immediately

Yun	1996	318	RCT	44–76	44–75	China	Isosorbide dinitrate	6–10# Q4–6 h	10# tid	ECG and symptom	2 weeks

Li	1998	154	RCT	58.93 ± 10.91	59.25 ± 10.12	China	Isosorbide dinitrate	0.2 g tid	10# tid	ECG and symptom	8 weeks

Feng and Zhou	2000	500	RCT	56 ± 4.24	55 ± 4.36	China	Salvia	10# tid	10# tid	ECG and symptom, Lipid profile and hemorheology	4 weeks

Mei	2000	128	RCT	NR	NR	China	Isosorbide dinitrate	6# tid	10# tid	ECG, symptom, and hemorheology	4 weeks

Xiao	2000	138	RCT	NR	NR	China	Isosorbide dinitrate	5# tid	10# tid	ECG and symptom	4 weeks

Ru	2000	90	RCT	45–70	NR	China	Salvia	5# tid	3# tid	ECG, symptom, lipid profile, and hemorheology	Unclear

Shu	2000	80	RCT	58.3 ± 7.24	57.2 ± 8.27	China	Isosorbide dinitrate	10# tid	10# tid	ECG and symptom	4 weeks

Yong	2000	148	RCT	45–72	47–70	China	Nitroglycerin	10#	5 mg	ECG, Symptom, blood pressure, and heart rate	Immediately

Guang	2000	248	RCT	NR	NR	China	Isosorbide dinitrate	5# tid	10# tid	Blood pressure, heart rate, and lipid profile	4 weeks

Li	2000	166	RCT	NR	NR	China	Nitroglycerin	5# tid	0.5 mg	ECG and symptom	2 weeks

Yu	2000	184	RCT	40–82	NR	China	Isosorbide dinitrate	5# tid	10# tid	ECG and symptom	15 days

Ya	2000	60	RCT	60–84	61–84	China	Nitroglycerin	5# tid	10# tid	ECG and symptom	4 weeks

Yuan	2000	102	RCT	61.68 ± 4.71	59.53 ± 5.62	China	Huoxin pills	5# tid	1# tid	ECG, symptom, and lipid profile	4 weeks

Duan and Yang	2002	80	RCT	42–79	41–75	China	Xinkeshu capsule	5# tid	4# tid	ECG, UCG, lipid profile, and hemorheology	4 weeks

Hai	2002	70	RCT	57 ± 7	59 ± 6	China	Isosorbide dinitrate	5# tid	10# tid	Symptom and hemorheology	4 weeks

Bu	2002	100	RCT	32–72	NR	China	Isosorbide dinitrate	4–6# tid	10–20# tid	ECG and symptom	Unclear

Pei	2003	102	RCT	56.1	55.2	China	Nitroglycerin	5# tid	Unclear	ECG, lipid profile, and hemorheology	8 weeks

Jin	2003	178	RCT	NR	NR	China	Isosorbide dinitrate	5# tid	20 mg qd	ECG, Symptom, lipid profile, and hemorheology	6 weeks

Ma	2004	116	RCT	63.4 ± 6.74	62.9 ± 7.84	China	Salvia	10# tid	3# tid	ECG, symptom, and hemorheology	4 weeks

Pei	2004	100	RCT	57.5 ± 10.2	63.1 ± 7.9	China	Xinkeshu capsule	6# tid	4# tid	ECG and symptom	4 weeks

Yue	2004	78	RCT	52–75	55–74	China	Placebo	5# tid	10# tid	ECG, UCG, and symptom	4 weeks

Hu	2004	80	RCT	51.56 ± 11.69	50.89 ± 11.02	China	Nitroglycerin	10# tid	0.5 mg tid	ECG and symptom	24 weeks

Zhu	2005	199	RCT	61.8	59.5	China	Glucose-insulin-potassium therapy	4–6# tid	Q2 d	ECG, symptom, and lipid profile	15 days

Wan	2005	64	RCT	NR	NR	China	Hesu pills	5# tid	1# tid	ECG and symptom	2 weeks

Shui	2006	73	RCT	43–78	45–76	China	Salvia	10# tid	10# tid	ECG and lipid profile	8 weeks

Cao and Zhang	2007	187	RCT	57.15 ± 5.38	58.77 ± 5.01	China	Salvia	6# tid	6# tid	ECG and symptom	2 weeks

Run	2007	90	RCT	NR	NR	China	Isosorbide dinitrate	10–15#	10 mg	ECG and symptom	Immediately

Wang	2008	60	RCT	63.9 ± 12.1	64.1 ± 11.2	China	Shexiang Baoxin pills	5# tid	1# tid	ECG and symptom	2 weeks

L. Wen	2009	88	RCT	56.7	56.8	China	Isosorbide dinitrate	5# tid	10# tid	ECG and symptom	8 weeks

Feng	2009	900	RCT	NR	NR	China	Salvia	6# tid	3# tid	ECG and symptom	4 weeks

S. L. Wen	2009	50	RCT	NR	NR	China	Tongxinluo pills	5# tid	4# tid	ECG and symptom	2 weeks

Guo	2012	60	RCT	42–70	43–73	China	Salvia	5# tid	3# tid	UCG and hemorheology	2 weeks

Qiao	2012	300	RCT	39–82	40–81	China	Salvia	5# tid	3# tid	ECG and symptom	4 weeks

Xin	2013	289	RCT	58.86 ± 10.57	57.69 ± 9.93	China	Standard therapy	10# tid	Blank	ECG and symptom	2 weeks

Long	2013	120	RCT	43–68	42–67	China	Standard therapy	10# tid	Blank	ECG and symptom	2 weeks

Li	2015	100	RCT	44–75	45–76	China	Salvia	6# tid	3# tid	ECG and symptom	4 weeks

NR: not reported; RCT: randomized controlled trial; tid: three times a day; Q4–6 h: every 4–6 h; Q8 h: every 8 h; ECG: electrocardiograph; UCG: ultrasound cardiogram; #: tablet.

**Table 2:** *Jadad* quality scores of selected studies.

Author name	Randomization	Generating randomized sequences	Blinding	Withdrawals and dropouts	Overall
Guan-hua He	1	0	0	0	1
Zhi-jin Song	1	0	0	0	1
Dong-ping Wang	1	0	1	0	2
Ke-fu Ji	1	0	0	0	1
Yu-chu Gao	1	0	0	0	1
Yun-yuan Guo	1	0	0	0	1
Li An	1	0	0	0	1
Ling Feng	1	0	0	0	1
Mei Hu	1	0	0	1	2
Xiao-chun Liu	1	0	0	0	1
Ru-bao Jia	1	0	0	0	1
Shu-dong Yang	1	0	0	0	1
Yong-jin Hou	1	0	0	0	1
Guang-yu Tang	1	0	0	0	1
Li-jun Zhou	1	0	0	0	1
Yu-ping Li	1	0	0	0	1
Ya-xiong Zhan	1	0	1	0	2
Jing-xian Yuan	1	0	0	0	1
Ke-jie Duan	1	0	0	0	1
Hai Shi	1	0	0	0	1
Bu-ce Sun	1	0	0	0	1
Pei-ying Wu	1	0	0	0	1
Jin Gao	1	0	0	0	1
Xian-zhen Ma	1	0	0	0	1
Pei-fen Chang	1	0	0	0	1
Yue-sheng Zhao	1	0	0	0	1
Gang Hu	1	0	0	0	1
Dong-you Zhu	1	0	0	0	1
Wei Wan	1	0	0	0	1
Shui-xiang Wan	1	0	0	0	1
Sheng-hai Cao	1	0	0	0	1
Run-lian Tang	1	0	0	1	2
Fei Wang	1	0	0	0	1
Wen Luo	1	0	0	0	1
Feng-hua Song	1	0	0	0	1
Wen-sheng Li	1	0	1	0	2
Wei-qin Guo	1	0	0	0	1
Qiao-kun Xu	1	0	0	0	1
Xin He	1	0	0	0	1
Long-jiang Qian	1	0	0	0	1
Li Xiao-jin	1	0	0	1	2

**Table 3:** Metaregression based on sample size and mean age.

Outcomes	Sample size	Mean age
Electrocardiogram improvement	0.206	0.059
Total cholesterol	0.758	0.236
Total triglycerides	0.013	0.507
Total low-density lipoprotein	0.283	0.513
Total high-density lipoprotein	0.715	0.789
Low-cutting whole blood Viscosity	0.917	0.774
High-cutting whole blood Viscosity	0.412	0.621
Plasma viscosity	0.075	0.842
Hematocrit	0.049	0.490
Fibrinogen	0.890	0.345
Adverse reactions	0.554	0.772

## Supplementary Materials

Supplementary Materials Figure S1: mean difference in total cholesterol in the treatment and control groups. Figure S2: mean difference in total triglycerides in the treatment and control groups. Figure S3: mean difference in total low-density lipoprotein in the treatment and control groups. Figure S4: mean difference in total high-density lipoprotein in the treatment and control groups. Figure S5: mean difference in low-cut whole blood viscosity in the treatment and control groups. Figure S6: mean difference in high-cut whole blood viscosity in the treatment and control groups. Figure S7: mean difference in plasma viscosity in the treatment and control groups. Figure S8: mean difference in hematocrit in the treatment and control groups. Figure S9: mean difference in the fibrinogen in the treatment and control groups. Figure S10: relative risks for adverse reactions in the treatment and control groups. Figure S11: funnel plot of studies with reported adverse reactions included in the meta-analysis.
