# Supplementary material for: Efficacy of Suxiao Jiuxin Pill on Coronary Heart Disease: A Meta-Analysis of Randomized Controlled Trials
Source: Evid Based Complement Alternat Med. 2018 Mar 27;2018:9745804. doi: 10.1155/2018/9745804 (PMC5892298; doi:10.1155/2018/9745804)
Supplement: Supplementary Materials — Figure S1: mean difference in total cholesterol in the treatment and control groups. Figure S2: mean difference in total triglycerides in the treatment and control groups. Figure S3: mean difference in total low-density lipoprotein in the treatment and control groups. Figure S4: mean difference in total high-density lipoprotein in the treatment and control groups. Figure S5: mean difference in low-cut whole blood viscosity in the treatment and control groups. Figure S6: mean difference in high-cut whole blood viscosity in the treatment and control groups. Figure S7: mean difference in plasma viscosity in the treatment and control groups. Figure S8: mean difference in hematocrit in the treatment and control groups. Figure S9: mean difference in the fibrinogen in the treatment and control groups. Figure S10: relative risks for adverse reactions in the treatment and control groups. Figure S11: funnel plot of studies with reported adverse reactions included in the meta-analysis. [file 9745804.f1.docx]

Figure S1. Mean difference in total cholesterol in the treatment and control groups

Figure S2. Mean difference in total triglycerides in the treatment and control groups

Figure S3. Mean difference in total low-density lipoprotein in the treatment and control groups

Figure S4. Mean difference in total high-density lipoprotein in the treatment and control groups

Figure S5. Mean difference in low-cut whole blood viscosity in the treatment and control groups

Figure S6. Mean difference in high-cut whole blood viscosity in the treatment and control groups

Figure S7. Mean difference in plasma viscosity in the treatment and control groups

Figure S8. Mean difference in hematocrit in the treatment and control groups

Figure S9. Mean difference in the fibrinogen in the treatment and control groups.

Figure S10. Relative risks for adverse reactions in the treatment and control groups

Figure S11. Funnel plot of studies with reported adverse reactions included in the meta-analysis
